# The Deficiency of SCARB2/LIMP-2 Impairs Metabolism via Disrupted mTORC1-Dependent Mitochondrial OXPHOS

**DOI:** 10.3390/ijms23158634

**Published:** 2022-08-03

**Authors:** Yujie Zou, Jingwen Pei, Yushu Wang, Qin Chen, Minli Sun, Lulu Kang, Xuyuan Zhang, Liguo Zhang, Xiang Gao, Zhaoyu Lin

**Affiliations:** 1 Ministry of Education Key Laboratory of Model Animal for Disease Study, State Key Laboratory of Pharmaceutical Biotechnology, Jiangsu Key Laboratory of Molecular Medicine, Model Animal Research Center, Medical School, Nanjing University, 12 Xuefu Road, Pukou Area, Nanjing 210061, China; zouyj@nicemice.cn (Y.Z.); peijw@nicemice.cn (J.P.); wangys@nicemice.cn (Y.W.); sunml@nicemice.cn (M.S.); kangll@nicemice.cn (L.K.); 2 Department of Oral Surgery, Shanghai Jiao Tong University, 639 Zhizaoju Road, Huangpu District, Shanghai 200240, China; chenqin@nicemice.cn; 3 The Center of Infection and Immunity, The Institute of Biophysics, Chinese Academy of Sciences, 15 Datun Road, Chaoyang District, Beijing 100101, China; zxy_better@ibp.ac.cn (X.Z.); liguozhang@ibp.ac.cn (L.Z.)

**Keywords:** LIMP-2 (SCARB2), lysosomes, mitochondria, OXPHOS, glycolysis, mTORC1/4E-BP1

## Abstract

Deficiency in scavenger receptor class B, member 2 (SCARB2) is related to both Gaucher disease (GD) and Parkinson’s disease (PD), which are both neurodegenerative-related diseases without cure. Although both diseases lead to weight loss, which affects the quality of life and the progress of diseases, the underlying molecular mechanism is still unclear. In this study, we found that *Scarb2^−/−^* mice showed significantly reduced lipid storage in white fat tissues (WAT) compared to WT mice on a regular chow diet. However, the phenotype is independent of heat production, activity, food intake or energy absorption. Furthermore, adipocyte differentiation and cholesterol homeostasis were unaffected. We found that the impaired lipid accumulation of *Adiponectin-cre; Scarb2^fl/fl^* mice was due to the imbalance between glycolysis and oxidative phosphorylation (OXPHOS). Mechanistically, the mechanistic target of rapamycin complex 1 (mTORC1)/ eukaryotic translation initiation factor 4E binding protein 1 (4E-BP1) pathway was down-regulated in Scarb2 deficient adipocytes, leading to impaired mitochondrial respiration and enhanced glycolysis. Altogether, we reveal the role of SCARB2 in metabolism regulation besides the nervous system, which provides a theoretical basis for weight loss treatment of patients with neurodegenerative diseases.

## 1. Introduction

Lysosomal integral membrane protein-2 (LIMP-2), also known as scavenger receptor class B, member 2 (SCARB2), is a receptor for the trafficking of glucocerebrosidase (GCase) from the endoplasmic reticulum to lysosomes [1]. Deficiency in SCARB2 has been found to be associated with Gaucher disease (GD) and Parkinson’s disease (PD), which are both neurodegenerative diseases [2,3,4,5]. Loss-of-function mutations of *SCARB2* could cause a decrease in lysosomal GCase activity with consequent accumulation of glucosylceramide and glucosylsphingosine in lysosomes of macrophages, which contributes to the phenotypic heterogeneity observed in GD [2,6,7]. SCARB2 also impairs the degradation of the fibril-forming protein α-synucleina-syn [8], potentially leading to a decrease in ceramide levels in PD [9,10].

Abnormal body weight and related metabolic disorders are common in both GD and PD patients, resulting in pathological changes, often with more severe effects on the development of the underlying disease and patient’s quality of life [11,12,13]. People used to think that weight loss was caused by a neurological problem, which could lead to low appetite [14,15]. However, some other reports suggest the weight loss is not all dependent on the nervous system [16,17,18]. Recently, SCARB2 is reported as a regulator of cholesterol metabolism in an in vitro system [19,20]. We wonder whether SCARB2 plays an important role in weight loss independent of the nervous system in vivo.

In this study, we showed that *Scarb2^−/−^* mice have significantly lower body weight than WT mice due to less WAT just on a regular chow diet. Using *Adiponectin-cre*; *Scarb2^f^*^l/fl^ mice, we further confirmed that the metabolic deficiency caused by Scarb2 depletion is not dependent on the nervous system. To identify the cause of weight loss, we checked the heat production, food intake, energy absorption, activity, adipocyte differentiation and cholesterol metabolism. However, all the biological progress above cannot explain the weight loss. Finally, we found that weight loss may be due to the imbalance between glycolysis and oxidative phosphorylation (OXPHOS) of adipocytes. Mechanistically, the activation of mTORC1 was disrupted on the surface of swollen lysosome as a consequence of Scarb2 deficiency, leading to decreased phosphorylation of mTORC1 and 4e-bp1 with reduced expression of mitochondrial transcription factor A (Tfam). Collectively, our study indicates that Scarb2 plays an important role in metabolism, independent of the nervous system.

## 2. Results

### 2.1. Scarb2^−/−^ Mice Show Less Lipid Accumulation

**Figure 1:**
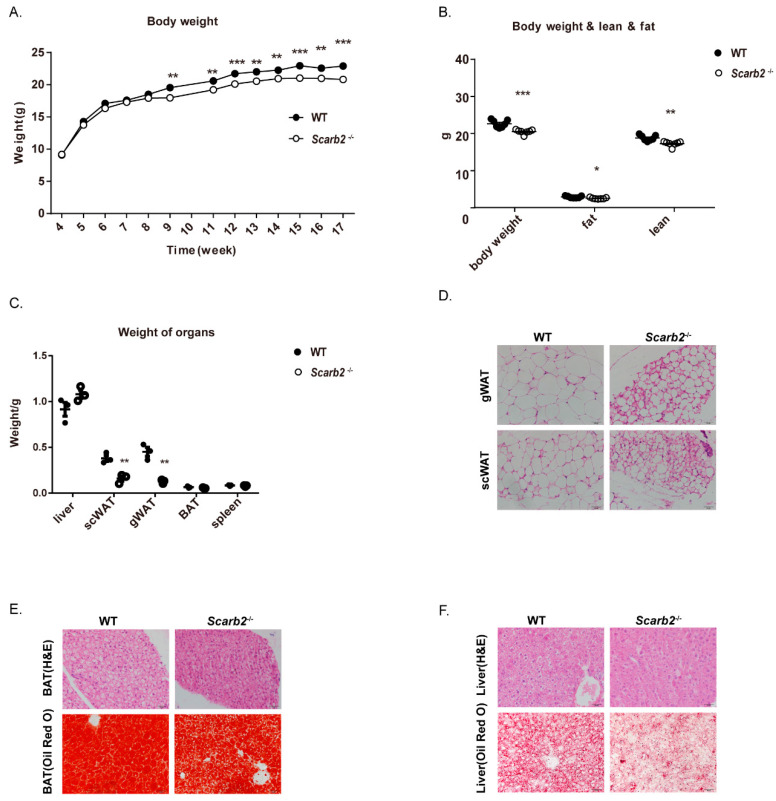
*Scarb2^−/−^* mice show less lipid accumulation on a regular chow diet. (**A**) Body weights of female WT and *Scarb2^−/−^* mice on a regular chow diet between 4–17 weeks. *n* = 7. (**B**) Body composition of female WT and *Scarb2^−/−^* mice measured by DEXA after 13 weeks on a regular chow diet. *n* = 7. (**C**) Weight of liver, gWAT, scWAT, BAT, spleen of 20-week-old female WT and *Scarb2^−/−^* mice (*n* = 3). (**D**) HE staining of gWAT and scWAT sections from 20-week-old female WT and *Scarb2^−/−^* mice. Scale bars, 20 μm. (**E**) HE and Oil Red O staining of BAT sections from 20-week-old female WT and *Scarb2^−/−^* mice. Scale bars, 20 μm. (**F**) HE and Oil Red O staining of liver sections from 20-week-old female WT and *Scarb2^−/−^* mice. Scale bars, 20 μm. DEXA: dual-energy X-ray absorptiometry, scWAT: subcutaneous white adipose tissue, gWAT: gonadal white adipose tissue, BAT: brown adipose tissue. Data are represented as mean ± SEM. * *p* < 0.05, ** *p* < 0.01, *** *p* < 0.001.

At first, the *Scarb2^−/−^* mice were generated by the CRISPR/Cas9 system which deletes 14bp in exon 1 and form a frame shift mutation (Figure S1A). The qPCR and Western blot results confirmed that *Scarb2* were not expressed in *Scarb2^−/−^* mice (Figure S1B,C). In this study, we found that both female and male *Scarb2^−/−^* mice showed a lower body weight than WT mice on regular chow diet (Figure 1A and Figure S1D), although the body length was comparable (Figure S1F–H). Furthermore, we analyzed the body composition by dual-energy X-ray absorptiometry (DEXA). Consistently, the female and male *Scarb2^−/−^* mice showed a lower body weight and less fat mass, which is similar to GD and PD patients [11,12,13] (Figure 1B and Figure S1E). Since the female *Scarb2^−/−^* mice showed body weight differences earlier than males, the following experiments were performed on females (Figure 1A and Figure S1D). The female *Scarb2^−/−^* mice also showed enhanced glucose tolerance relative to the control mice, but insulin sensitivity was comparable (Figure S1I,J).

To investigate the reason for fat mass loss, we weighted livers and adipose tissues at first. The results showed that the loss of fat mass was mainly due to less WAT (Figure 1C and Figure S1K). Consistently, gonadal and subcutaneous adipocytes were smaller in *Scarb2^−/−^* mice (Figure 1D). Although the weight of liver or brown adipose tissue (BAT) showed no significant difference between *Scarb2^−/−^* mice and control mice, lipid accumulation of *Scarb2^−/−^* mouse liver and BAT was impaired (Figure 1E,F). Together, these results suggest that *Scarb2^−/−^* mice have less lipid accumulation compared to the control mice.

### 2.2. The Less Lipid Accumulation in Scarb2^−/−^ Mice Is Independent of Heat Production, Activity, Food Intake and Energy Absorption

**Figure 2:**
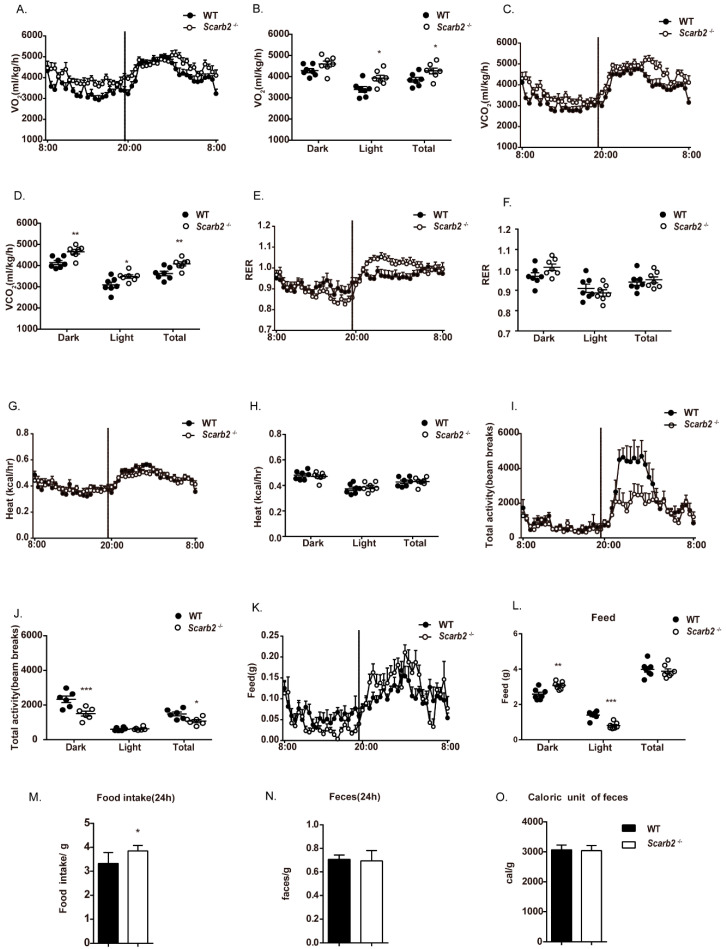
Metabolic phenotype analysis of female *Scarb2^−/−^* mice on a regular chow diet. (**A**–**L**) Indirect calorimetry of 16-week-old female WT and *Scarb2^−/−^* mice: (**A**,**B**) VO_2_: oxygen consumption. (**C**,**D**) VCO_2_: CO_2_ generation. (**E**,**F**) RER: respiration exchange rate, VCO_2_/VO_2._ (**G**,**H**) HEAT: heat generation. (**I**,**J**) Total activity. (**K**,**L**) FEED: food intake. *n* = 7. The line chart represents real time value of 24 h for 2 day average. The column chart represents an average value during the light cycle (8:00~20:00) and dark cycle (20:00~8:00). (**M**) Food consumption over 24 h of 8-week-old WT and *Scarb2^−/−^* mice (data represent the average of three days). *n* = 6 per genotype. (**N**) Faces weight over 24 h of 8-week-old WT and *Scarb2^−/−^* mice. *n* = 6 per genotype. (**O**) Assessment of energy harvest in 8-week-old WT and *Scarb2^−/−^* mice fed regular chow diet using fecal bomb calorimetry. *n* = 6 per genotype. Data are represented as mean ± SEM. * *p* < 0.05, ** *p* < 0.01, *** *p* < 0.001.

To identify the underlying mechanisms for less lipid accumulation in *Scarb2^−/−^* mice, we quantified changes in energy expenditure of WT and *Scarb2^−/−^* mice by metabolic cages. Female *Scarb2^−/−^* mice showed a higher oxygen (O_2_) consumption in both light and total time and a higher carbon dioxide (CO_2_) production throughout the day (Figure 2A–D). However, the respiration exchange rate (RER) and heat production showed no significant differences between the two groups, suggesting that the fat mass loss is not due to heat production change (Figure 2E–H). Furthermore, the total activity of female *Scarb2^−/−^* mice declined dramatically in the night as well as on the total level, suggesting that the reason for fat mass loss is not the activity (Figure 2I,J). All the results above suggest that energy expenditure increased in *Scarb2^−/−^* mice, but not through heat production or activity. The results of indirect calorimetry analysis of male *Scarb2^−/−^* mice were similar to female mice (Figure S2).

Interestingly, we found that the night food intake of *Scarb2^−/−^* mice showed a significant increase, while the daily food intake showed a significant decrease. However, there was no significant difference in the overall food intake tested by metabolic cages (Figure 2K,L). To confirm this result, the daily food consumption was investigated and calculated separately. It is surprising that *Scarb2^−/−^* mice even had more food consumption than the control, indicating that the fat mass loss is not due to food intake (Figure 2M). However, the differences in night food intake and daily food intake suggest a circadian change in *Scarb2^−/−^* mice.

We next checked whether deficiency of Scarb2 influences energy absorption. Fecal samples were collected and lyophilized. The dry mass of feces of *Scarb2^−/−^*mice was similar with the control mice (Figure 2N). Gross energy content of feces measured by semimicro oxygen bomb showed no difference, suggesting that Scarb2 is not involved in the absorption function (Figure 2O). Altogether, the impaired lipid accumulation in *Scarb2^−/−^* mice may be due to higher energy expenditure, but is independent of heat production, food intake, energy absorption or excessive activity.

### 2.3. The Less Lipid Accumulation in Scarb2^−/−^ Mice Is Independent of Cholesterol Metabolism

LIMP2/SCARB2 was recently reported to bind and deliver exogenous cholesterol to the lysosomal membrane and later to lipid droplets. Depletion of LIMP-2 alters sterol regulatory element binding protein (SREBP)-2-mediated cholesterol regulation, as well as low density lipoprotein (LDL)-receptor levels [19,20]. Glucocorticoids is an important growth hormone synthesized from cholesterol [21]. Cholesterol deficiency will decrease the glucocorticoid level, which may trigger symptoms of disturbed health status. So, we checked whether the deletion of Scarb2 impaired cholesterol metabolism or glucocorticoid levels. However, the level of cholesterol and corticosterone (Figure S3A,B) in plasma of *Scarb2^−/−^* mice had no differences with control mice. Besides, the transcript level of sterol regulatory element- binding protein 2 (*Srebp2*) and 3-hydroxy-3-methylglutaryl coenzyme A reductase (*Hmgcr*) in *Scarb2^−/−^* mouse liver, which are crucial players of the cholesterol biosynthetic pathway, are comparable to WT mice. Furthermore, we found no expression changes of key enzymes and regulators of glucocorticoid pathways (Figure S3C,D). Altogether, the results suggest that the overall cholesterol homeostasis is normal in *Scarb2^−/−^* mice.

### 2.4. The Deficiency of Scarb2 in Adipocytes Contribute to the Less Lipid Accumulation 

**Figure 3:**
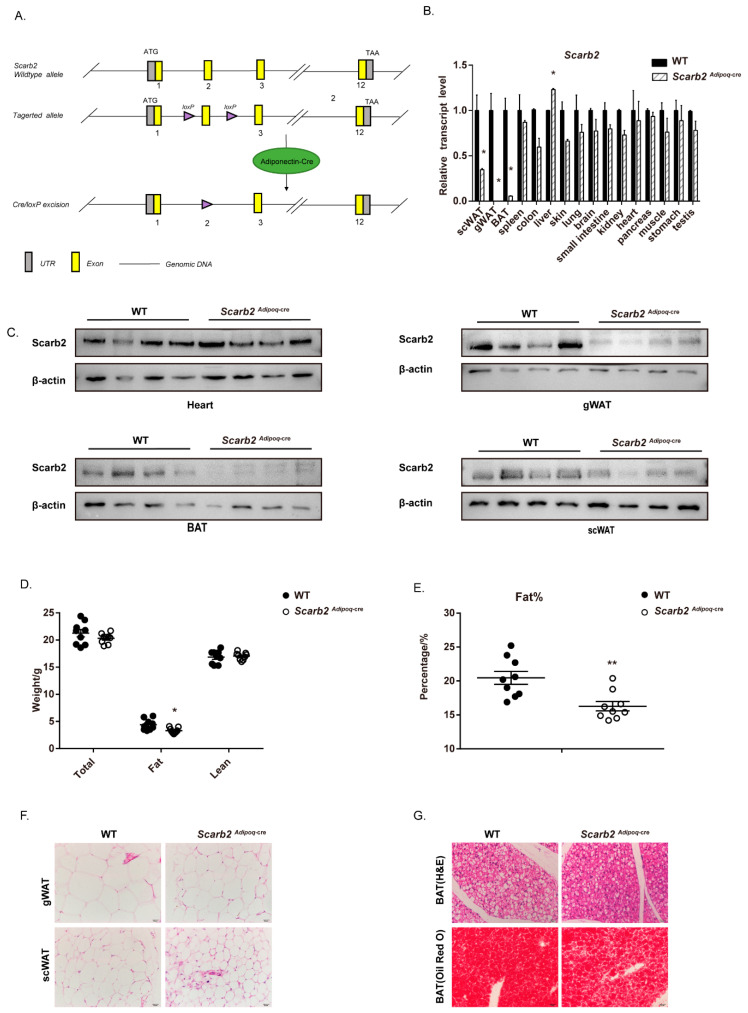
*Scarb2^Adipoq-cre^* mice show lower lipid accumulation. (**A**) Schematic representation of the *Scarb2* (**top**), and targeted gene (**bottom**). (**B**) The expression of *Scarb2* in different tissues and organs from *Scarb2^Adipoq-cre^* mice was analyzed by real-time quantitative PCR. Expression levels of target genes were normalized to *Rplp0* (alias *36B4*) and data presented in (**B**) were normalized to the mean value of WT group for each tissue or organ. *n* = 3. (**C**) Immunoblot analysis of Scarb2 and β-actin in heart, BAT, gWAT and scWAT from WT and *Scarb2^Adipoq-cre^* mice. *n* = 4. (**D**,**E**) Body composition and percentage of fat mass of female WT and *Scarb2^Adipoq-cre^* mice measured by DEXA after 12 weeks on a regular chow diet. *n* = 9. (**F**) HE staining of gWAT, scWAT and BAT sections from 12- week-old female WT and *Scarb2^Adipoq-cre^* mice. Scale bars, 20 μm. (**G**) HE and Oil Red O staining of BAT sections from 12- week-old female WT and *Scarb2^Adipoq-cre^* mice. Scale bars, 20 μm. DEXA: dual-energy X-ray absorptiometry, scWAT: subcutaneous white adipose tissue, gWAT: gonadal white adipose tissue, BAT: brown adipose tissue. Data are represented as mean ± SEM. * *p* < 0.05, ** *p* < 0.01.

Although a combination of various factors, such as hormones, rhythm, nervous system and muscles can affect body weight and lipid metabolism, we would like to focus on the role of SCARB2 in adipose tissue. To rule out the influence of other tissues or systems on fat mass, especially nervous systems, we generated *Adiponectin-cre*; *Scarb2^f^*^l/fl^ mice (*Scarb2^Adipoq-cre^* mice) (Figure 3A). In *Scarb2^Adipoq-cre^* mice, Exon 2 of *Scarb2* is floxed by loxP sites and removed via Adiponectin-cre only in adipose tissue. The qPCR and Western blot results confirmed that Scarb2 were specifically deleted in mouse adipose tissue (Figure 3B,C). The *Scarb2^Adipoq-cre^* mice also showed lower fat mass compared to WT mice (Figure 3D,E and Figure S4A). Consistent with the *Scarb2^−/−^* mice, gonadal and subcutaneous adipocytes were smaller in *Scarb2^Adipoq-cre^* mice than in WT mice (Figure 3F). Less lipid accumulation was observed in WAT and BAT of *Scarb2^Adipoq-cre^* mice (Figure 3F,G). These results demonstrate that the depletion of Scarb2 in adipocytes is enough to impair the lipid accumulation.

### 2.5. The Less Lipid Accumulation Is Independent of Adipocyte Differentiation 

**Figure 4:**
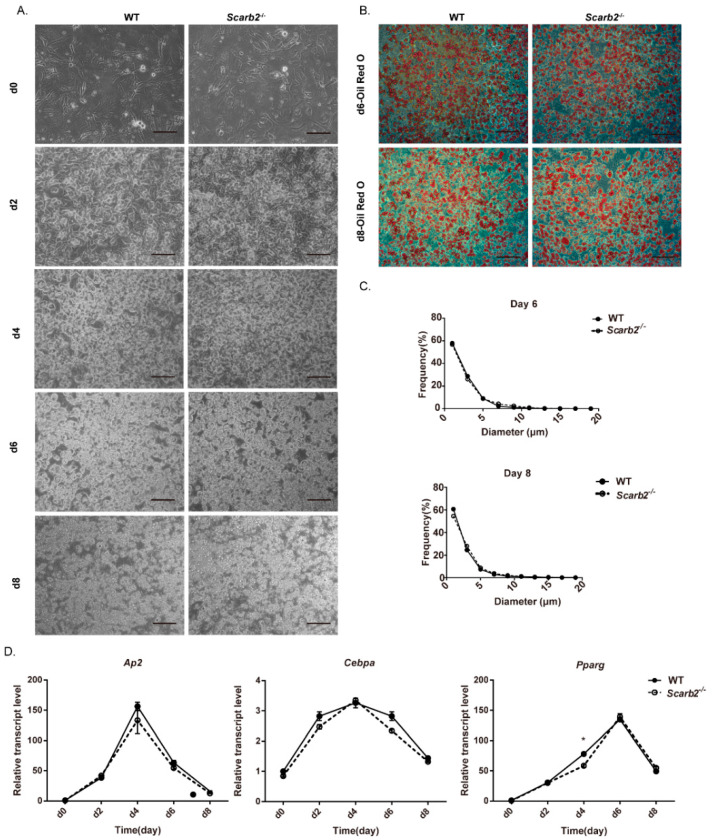
SCARB2 is not required for adipocyte differentiation. (**A**) Pictures of differentiated preadipocytes from 10-day-old female WT and *Scarb2^−/−^* mice on day 0, 2, 4, 6 and 8. Scale bars, 100 μm. (**B**) Oil Red O staining of differentiated preadipocytes on day 6 and day 8. Scale bars, 100 μm. (**C**) Statistics of the diameter of adipocytes stained with Oil Red O on day 6 and day 8 from WT and *Scarb2^−/−^* mice. *n* = 3. (**D**) Adipocyte markers *Ap2*, *C/ebpα* and *Pparγ* of differentiated preadipocytes were analyzed by real-time quantitative PCR. Expression levels of target genes were normalized to *Rplp0* (alias *36B4*) and data presented in (**D**) were normalized to the day 0 value of each experiment. *n* = 3. Data are shown as means ± SEM. * *p* < 0.05.

To check whether Scarb2 affects adipocyte differentiation, pre-adipocytes from the *Scarb2^−/−^* mice and control mice were differentiated into adipocytes in vitro as previous reports [22,23]. Oil Red O staining showed that there was no difference in adipocyte differentiation between wild-type and *Scarb2^−/−^* groups (Figure 4A–C). Furthermore, the mRNA levels of specific adipocyte differentiation markers, such as *Pparγ*, *C/ebpα* and *Ap2*, were comparable between *Scarb2^−/−^* and control groups during the whole process (Figure 4D). All the results above suggest that the less lipid accumulation in *Scarb2^Adipoq-cre^* mice is independent of adipocyte differentiation.

### 2.6. Enhanced Glycolysis and Impaired Oxidative Phosphorylation (OXPHOS) in Scarb2^−/−^ Adipocytes

**Figure 5:**
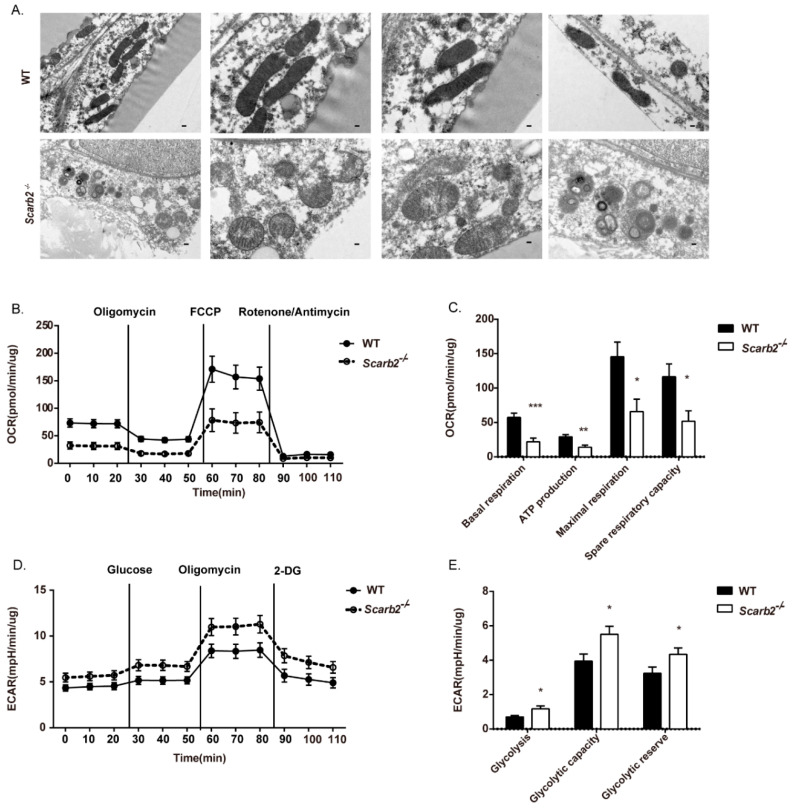
Enhanced glycolysis and impaired oxidative phosphorylation (OXPHOS) in *Scarb2^−/−^* adipocytes. (**A**) Representative TEM images of WT and *Scarb2^−/−^* WAT from 12- week-old female mice. Scale bar = 0.2 μm in the first column. Scale bar = 0.1 μm in the others. (**B**) Real-time changes in OCR (a measure of oxidative phosphorylation) of WT or *Scarb2^−/−^* adipocytes measured by Seahorse XF24 Extracellular Flux Analyzer. The compounds (oligomycin, FCCP, and a mix of rotenone and antimycin A) are serially injected to measure ATP production, maximal respiration, and non-mitochondrial respiration, respectively. *n* = 5. (**C**) Assessment of basal OCR, ATP production, spare and maximal respiratory capacity of adipocytes in (**B**). (**D**) Real-time changes in ECAR (a measure of lactate production and glycolysis) of WT or *Scarb2^−/−^* adipocytes measured by Seahorse XF24 Extracellular Flux Analyzer. The compounds (glucose, oligomycin and 2-DG) are serially injected to measure glycolysis, glycolytic capacity, and non-glycolytic acidification, respectively, which also allows calculation of glycolytic reserve. *n* = 5. (**E**) Assessment of glycolysis, glycolytic capacity and glycolytic reserve of adipocytes in (**D**). OCR: oxygen consumption rate, ECAR: extracellular acidification rate, FCCP: Carbonyl cyanide 4-(trifluoromethoxy) phenylhydrazone. ATP: Adenosine triphosphate. * *p* < 0.05, ** *p* < 0.01, *** *p* < 0.001.

Mitochondria are the most important place for OXPHOS and Adenosine triphosphate (ATP) production. Several reports have demonstrated impaired mitochondrial respiration in multiple Gaucher’s models [24,25,26,27]. Thus, we investigated mitochondrial morphology by transmission electron microscopy (TEM) and found that mitochondria was impaired in *Scarb2^−/−^* adipocytes (Figure 5A). Elongated mitochondria with a dark dense matrix were observed in WT WAT (Figure 5A, the upper row). In contrast, swollen mitochondria with low-density internal staining were observed in *Scarb2^−/−^* WAT (Figure 5A, the bottom row).

Next, we analyzed the metabolic function of adipocytes from the *Scarb2^−/−^* mice and control mice by Seahorse. *Scarb2^−/−^* adipocytes showed significantly lower basal respiration and ATP production (Figure 5B,C), indicating impaired mitochondrial respiration. The level of glycolysis were significantly elevated in *Scarb2^−/−^* adipocytes (Figure 5D,E). The mitochondrial respiration is considered to be a more efficient metabolic process for ATP synthesis in comparison to glycolysis. It suggests that the imbalance between glycolysis and OXPHOS may lead to extra energy and nutrient expenditure and less lipid accumulation.

### 2.7. Abnormal Lysosomes and Dysregulated mTORC1 Pathway in Scarb2^−/−^ Adipocytes

**Figure 6:**
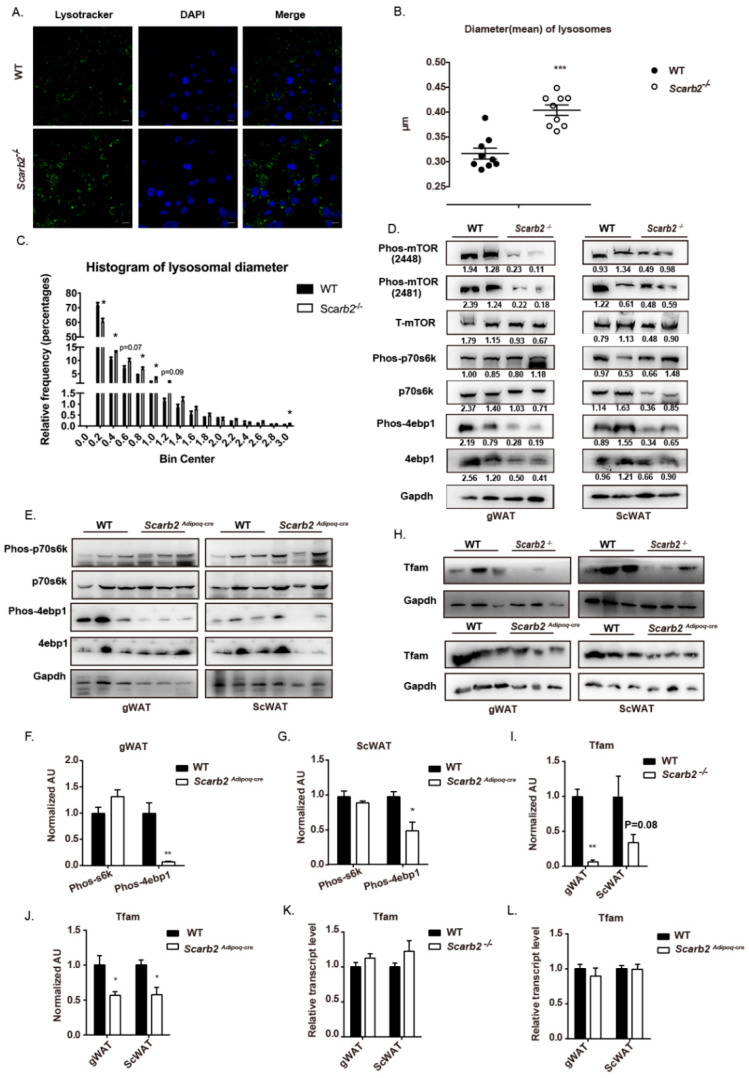
mTORC1 pathway is impaired in *Scarb2^−/−^* and *Scarb2^Adipoq^*^-cre^ WAT. (**A**) Immunofluorescence of mature adipocytes differentiated from pre-adipocytes of WT and *Scarb2^−/−^* mice stained with Lysotracker DND-26 (green) and DAPI (blue). Scale bars, 10 μm. (**B**) The statistical result of mean diameter of lysosomes in pre-adipocytes of WT and Scarb2^−/−^ mice. *n* = 9. (**C**) Histogram of diameter of lysosomes in pre-adipocytes of WT and Scarb2^−/−^ mice. *n* = 9. (**D**) Immunoblot analysis of Phos-mTOR (2448 and 2481), Total-mTOR (T-mTOR), Phos-p70s6k, p70s6k, Phos-4ebp1, 4ebp1 and Gapdh in WT and *Scarb2^−/−^* mouse WAT was shown. The value of gray scale analysis were calculated by Image J and labeled below bands. Phos-mTOR (2448), Phos-mTOR (2481), Phos-p70s6k and Phos-4ebp1 were normalized to the respective total protein, p70s6k, T-mTOR and 4ebp1 were normalized to GAPDH. (**E**) Immunoblot analysis of Phos-p70s6k, p70s6k, Phos-4ebp1, 4ebp1 and Gapdh in gWAT or scWAT of WT and *Scarb2^Adipoq^*^-cre^ mice was shown. (**F**,**G**) The gray scale analysis of phos-s6k/s6k and phos-4e-bp1/4e-bp1 in (**E**). All the data were normalized to the mean value of phos-s6k/s6k or phos-4e-bp1/4e-bp1 in WT group, respectively. (**H**) Immunoblot analysis of Tfam and Gapdh in gWAT or scWAT of WT, *Scarb2^−/−^* and *Scarb2^Adipoq^*^-cre^ mice was shown. (**I**,**J**) The gray scale analysis of Tfam /Gapdh in (**H**). All the data were normalized to the mean value of Tfam /Gapdh in WT group. (**K**) The mRNA levels of *Tfam* in WAT of WT and *Scarb2^−/−^* mice analyzed by real-time quantitative PCR. *n* = 4. Expression levels of target genes were normalized to *Rplp0* (alias *36B4*). All the data were normalized to the mean value of WT group. (**L**) The mRNA levels of *Tfam* in WAT of WT and *Scarb2^Adipoq^*^-cre^ mice analyzed by real-time quantitative PCR. *n* = 4. Expression levels of target genes were normalized to *Rplp0* (alias *36B4*). All the data were normalized to the mean value of WT group. scWAT: subcutaneous white adipose tissue, gWAT: gonadal white adipose tissue. Data are shown as means ± SEM. * *p* < 0.05, ** *p* < 0.01, *** *p* < 0.001.

The mitochondrial dysfunction is often correlated with lysosomal dysfunction [28]. As reported, the deficiency of Scarb2 will lead to the morphological change in lysosomes. First, we confirmed whether the morphology of lysosomes was impaired in *Scarb2^−/−^* adipocytes. The lysosomes in *Scarb2^−/−^* adipocytes were bigger than those in WT (Figure 6A,B), which were similar to the swollen lysosomes in other lysosomal storage disorders [29]. In addition, frequency distribution of lysosomal diameter showed that the percentages of big lysosomes in *Scarb2^−/−^* adipocytes were much higher than those of WT (Figure 6C).

In addition to their role in intracellular degradation, lysosomes also modulate mechanistic Target of Rapamycin (mTOR) complex 1, a classic cell-signaling which contributes to the metabolic balance between anabolism and catabolism [30,31]. Consistently, we found that the mTORC1 pathway was dysregulated in *Scarb2^−/−^* mice. The phosphorylation of Ser2448 and Ser2481 of mTORC1 were decreased in WAT of *Scarb2^−/−^* mice (Figure 6D), indicating the activity of mTORC1 is impaired. Furthermore, the key downstream signal of mTORC1, the phosphorylated 4e-bp1 (canonical substrates eukaryotic translation initiation factor 4E (eIF4E)-binding protein1) decreased in WAT of both *Scarb2^−/−^* and *Scarb2^Adipoq-cre^* mice (Figure 6D–G). mTORC1 pathways regulated the translation of TFAM, which is one of key mitochondrial genes needed for oxidative phosphorylation [32,33,34]. The protein levels of Tfam were decreased both in *Scarb2^−/−^* and *Scarb2^Adipoq^*^-cre^ WAT without the changes of mRNA levels (Figure 6H–L). It indicates that enhanced glycolysis and decreased OXPHOS are induced by the impaired mTORC1 pathway.

**Figure 7:**
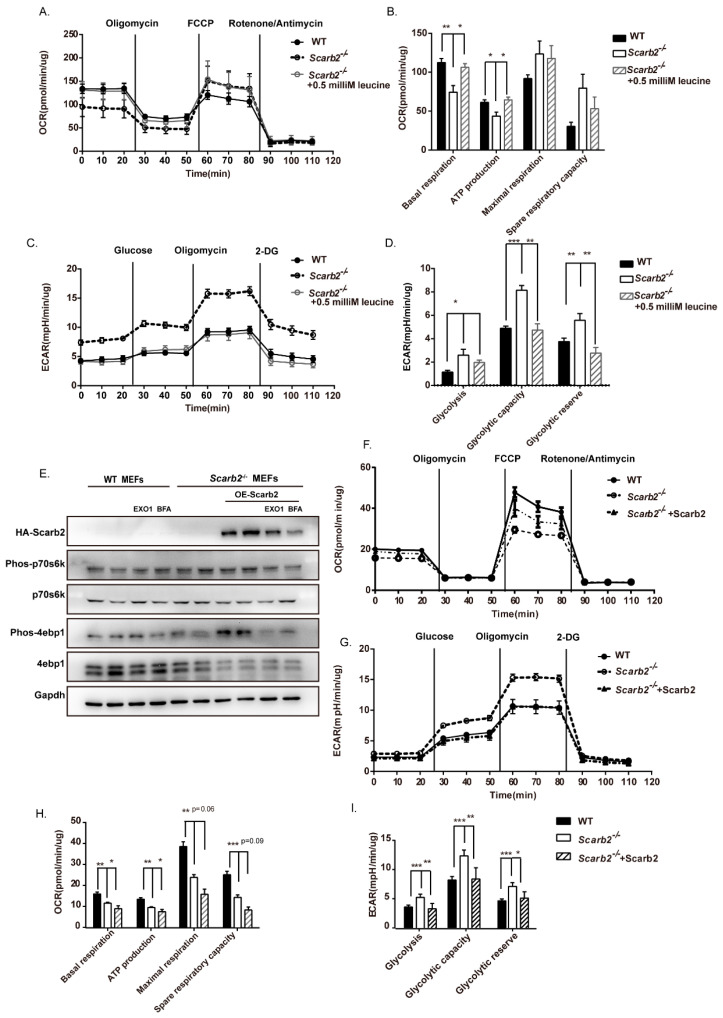
Activation of mTORC1 rescues impaired OXPHOS in *Scarb2^−/−^* adipocytes. (**A**) Real-time changes in OCR (a measure of oxidative phosphorylation) of WT, *Scarb2^−/−^* adipocytes and *Scarb2^−/−^* adipocytes treated with 0.5 mM Leucine measured by Seahorse XF24 Extracellular Flux Analyzer. The compounds (oligomycin, FCCP and a mix of rotenone and antimycin A) are serially injected to measure ATP production, maximal respiration, and non-mitochondrial respiration, respectively. *n* = 5. (**B**) Assessment of basal OCR, ATP production, spare and maximal respiratory capacity of adipocytes in (**A**). (**C**) Real-time changes in ECAR (a measure of lactate production and glycolysis) of WT, *Scarb2^−/−^* adipocytes and *Scarb2^−/−^* adipocytes treated with 0.5 mM Leucine measured by Seahorse XF24 Extracellular Flux Analyzer. The compounds (glucose, oligomycin, and 2-DG) are serially injected to measure glycolysis, glycolytic capacity, and non-glycolytic acidification, respectively, which allows calculation of glycolytic reserve. *n* = 5. (**D**) Assessment of glycolysis, glycolytic capacity and glycolytic reserve of adipocytes in (**C**). For *Scarb2^−/−^* +0.5 mM Leucine group: 0.5 mM Leucine (final concentration) was added in Seahorse media, as the activator of the mTORC1 pathway. *Scarb2^−/−^* + 0.5 mM adipocytes were also pre-treated with 0.5 mM Leucine in media (final concentration) for 24 h before the Seahorse experiments. (**E**) Immunoblot analysis of HA, Phos-p70s6k, p70s6k, Phos-4ebp1, 4ebp1 and Gapdh in WT and *Scarb2^−/−^* MEFs are shown. Scarb2 was overexpressed in *Scarb2^−/−^* MEFs by transfecting plasmid pCDNA5-HA-Scarb2. EXO1 and Brefeldin A (BFA) are two inhibitors of protein trafficking. 90 microM EXO1 (final concentration) and 5 microM BFA (final concentration) was added in media respectively for 24 h before harvest. (**F**) Real-time changes in OCR (a measure of oxidative phosphorylation) of WT, *Scarb2^−/−^* MEFs and *Scarb2^−/−^* MEFs overexpressed Scarb2 measured by Seahorse XF24 Extracellular Flux Analyzer. The compounds (oligomycin, FCCP, and a mix of rotenone and antimycin A) are serially injected to measure ATP production, maximal respiration, and non-mitochondrial respiration, respectively. *n* = 5. (**G**) Real-time changes in ECAR (a measure of lactate production and glycolysis) of WT, *Scarb2^−/−^* MEFs and *Scarb2^−/−^* MEFs overexpressed Scarb2 measured by Seahorse XF24 Extracellular Flux Analyzer. The compounds (glucose, oligomycin, and 2-DG) are serially injected to measure glycolysis, glycolytic capacity, and non-glycolytic acidification, respectively, which allows calculation of glycolytic reserve. *n* = 5. (**H**) Assessment of basal OCR, ATP production, spare and maximal respiratory capacity of MEFs in (**F**). (**I**) Assessment of glycolysis, glycolytic capacity and glycolytic reserve in MEFs were calculated based on (**G**). For *Scarb2^−/−^* +Scarb2 group: Scarb2 was overexpressed in *Scarb2^−/−^* MEFs by transfecting plasmid pCDNA5-HA-Scarb2. MEFs: mouse embryonic fibroblast cells. OCR: oxygen consumption rate, ECAR: extracellular acidification rate, FCCP: Carbonyl cyanide 4-(trifluoromethoxy) phenylhydrazone, ATP: Adenosine triphosphate. * *p* < 0.05, ** *p* < 0.01, *** *p* < 0.001.

To confirm our results, we activated the mTORC1 pathway by leucine in adipocytes. We found that the activation of mTORC1 in *Scarb2^−/−^* adipocytes rescued the decreased OXPHOS and increased glycolysis level (Figure 7A–D). As primary adipocytes are not suited to the overexpression system, we overexpressed Scarb2 in *Scarb2^−/−^* mouse embryonic fibroblasts (MEFs), which could differentiate to adipocytes. We found that overexpressed Scarb2 rescued the level of phos-4e-bp1 (Figure 7E) and the levels of OXPHOS and glycolysis (Figure 7F–I). In addition, EXO1 and Brefeldin A (BFA), two inhibitors of protein trafficking which could mimic the deficiency of Scarb2, down-regulated the level of phos-4e-bp1, confirming the disruption of protein trafficking to lysosome can impair the activation of the mTORC1 pathway (Figure 7E). Altogether, our results suggest that the deficiency of Scarb2 leads to the impaired OXPHOS and increased glycolysis through mTORC1 pathways.

## 3. Discussion

**Figure 8:**
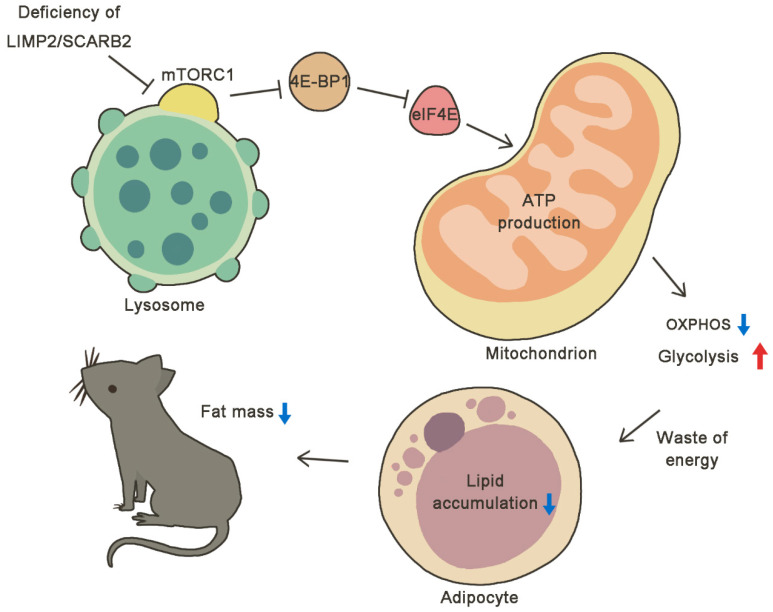
Model graph proposing the changes of lysosomes and mitochondria brought by the absence of SCARB2. The normal activation of mTORC1 was disrupted at the surface of swollen lysosome as a consequence of Scarb2 deficiency, leading to changes in the mTORC1 pathway with decreased phosphorylation of mTORC1 and 4E-BPs. Thus, the expression of Tfam was down-regulated and the ability to produce ATP by mitochondrial oxidative phosphorylation was reduced. The impaired OXPHOS and increased glycolysis leads to the waste of energy, resulting in less lipid accumulation and less fat mass in *Scarb2^−/−^* mice.

In this study, we found that the body weight of *Scarb2^−/−^* was much lower than control mice on a regular chow diet due to reduced fat mass. We found that the loss of body weight was not due to heat production or energy absorption. For calorie intake and activity, the *Scarb2^−/−^* mice ate even more and moved less than the WT mice, which is opposite to the increased energy consumption, suggesting Scarb2 may affect metabolism directly. To explore the independent role of Scarb2 in metabolism, we constructed *Adiponectin-cre; Scarb2^fl/fl^* mice and confirmed that the deficiency of Scarb2 in adipose tissue was enough to cause abnormal lipid accumulation. However, the adipocyte differentiation was unaffected in *Scarb2^−/−^* adipocytes. Furthermore, we analyzed the mitochondrial morphology and metabolic phenotype of *Scarb2^−/−^* WAT and uncovered the imbalance between glycolysis and OXPHOS. Mechanistically, we found that the activation of mTORC1 was disrupted at the surface of swollen lysosome as a consequence of Scarb2 deficiency, leading to decreased phosphorylation of mTORC1 and 4e-bp1 (Figure 8). Our study suggests that Scarb2 plays an important role, independent of the nervous system, in metabolism.

Although both *Scarb2^Adipoq-cre^* mice and *Scarb2^−/−^* mice showed a lower fat mass compared to the control mice, the degree of phenotype in the lipid accumulation of *Scarb2^Adipoq-cre^* mice was milder compared to *Scarb2^−/−^* mice. Furthermore, *Scarb2^−/−^* mice showed increased oxygen consumption (Figure 2B). It is inconsistent with the decrease in OXPHOS in *Scarb2^−/−^* adipocytes. The different phenotype may be due to dysregulated *Scarb2^−/−^* nervous system. *Scarb2^−/−^* mice have neurological abnormalities, and the nervous system may affect the body’s overall metabolism in some ways other than appetite or physical activity level, such as circadian rhythm. The decreased activity and increased food intake of *Scarb2^−/−^* mice in night may suggest the disruption of circadian rhythm in *Scarb2^−/−^* mice. The disruption of circadian rhythms may lead to the changes in metabolism and even diseases [35,36,37]. Food is the most important external signal to our peripheral clocks. Studies performed either in humans or mice suggest that the timing of food intake is relevant for obesity [38,39]. People who eat late in the evening were more likely to be obese and had less ability to lose weight [40]. It was also reported that mice fed with a high fat diet only during the dark weigh significantly less than mice fed only during the light [41], which corresponds to the phenotype of *Scarb2^−/−^* mice in our study. Abnormal sleep/wake patterns also have the potential to disrupt the circadian clocks and influence metabolism [42]. It was reported that the rates of lipolysis increase while an animal sleeps, resulting in increased release of non-esterified fatty acids. In turn, the rates of lipogenesis increase when an animal is awake [43]. It is consistent with the phenotype of *Scarb2^−/−^* mice in our study. Thus, the role of circadian rhythm in the metabolism of *Scarb2^−/−^* mice is worth investigation in the future.

Female *Scarb2^−/−^* mice showed earlier and more significant body weight decrease compared to the male mice. We monitored the muscular development of *Scarb2^−/−^* mice by grip strength test. The grasping ability of male *Scarb2^−/−^* mice showed a decreasing tendency without significance, while both the grasping ability of female *Scarb2^−/−^* mice forelimbs and limbs were significantly lower than WT mice (Figure S4B). It is consistent with previous data that female *Scarb2^−/−^* mice showed less lean mass, but not males (Figure 1B and Figure S1E). Sex differences exist in metabolic phenotypes mainly due to sex hormones, such as feeding behavior, fat distribution, energy expenditure and physical activity [44,45]. It has long been known that estrogens play an essential role in inhibiting feeding in females as there exists a reduction in food intake when estradiol presents its highest levels. The female mice are also intrinsically more variable than males for the estrous cycle [46]. On the contrary, progesterone and testosterone may stimulate appetite [47,48]. Exercise physiology is definitely different in males and females due to sex difference. Estrogen regulates fat distribution by acting at hypothalamus and adipose tissue level. Males have less total fat and more gonadal/central fat distribution while women have more total fat and more gluteal/ subcutaneous fat distribution [49,50]. The varying degrees of differences between male and female *Scarb2^−/−^* mice needs further investigation.

The mitochondrial dysfunction has been found in multiple lysosomal storage diseases (LSDs), including Niemann–Pick disease type C and Pompe disease [51]. Furthermore, the onset of neurodegenerative diseases (i.e., Parkinson’s disease, Alzheimer’s disease) is tightly linked to mutations in mitochondria or mitochondrial defects [52,53,54]. Several researchers have demonstrated impaired mitochondrial respiration in multiple models of Gaucher’s disease, the most common being LSD [24,25,26,27]. It is suggested that mitochondrial defects may be a common theme across LSDs and neurodegenerative diseases and may be partially mediated by defective mitochondria–lysosome mutual function secondary to lysosomal dysfunction. In this study, we found that the mTORC1 was dysregulated in *Scarb2^−/−^* mice with a decreased level of phos-4e-bp1, which is crucial for translation and mitochondrial normal function.

The mTORC1 signaling is one of the most important intracellular pathways that integrates dynamic environmental cues and regulates multitudinous metabolic pathways, including protein/lipid/nucleotide syntheses and glucose/glutamine/lipid/amino acid metabolism [55,56]. In our study, the cells with Scarb2 deficiency showed swollen and abnormal lysosomes. As reported, the recruitment of mTORC1 to the lysosomal surface has been shown to be essential for its activation [57,58]. The recruitment of mTORC1 to the lysosomal membrane is regulated by the Rag GTPases, which is composed of RagA or RagB and RagC or RagD. Then, when mTORC1 encounters its master activator, Rheb, which also resides on the lysosome, it will be activated by GTP-bound Rheb and starts to phosphorylate downstream substrates [59,60,61]. Thus, mechanistically, the activation of mTORC1 may be disrupted on the surface of swollen lysosome as a consequence of Scarb2 deficiency, leading to decreased phosphorylation of mTORC1 and 4e-bp1 with reduced expression of mitochondrial transcription factor A (Tfam). However, the activation of mTORC1 signaling is a process which is subjected to sophisticated and complex regulation [62]. The failure of any process during assemble of complexes and coordination may disrupt the normal activation of mTORC1 signal. The detail mechanisms that how SCARB2 could mediate mTORC1 by swollen lysosomes need further research.

4E-BP1 belongs to a family of translation repressor proteins, which consists of three small peptides. It is one of the two most established substrates of the mTORC1 signaling pathway that serves as key effector for protein synthesis [63]. The clinical blood chemistry analysis carried out on WT and *Scarb2^−/−^* mice showed that the level of blood urea nitrogen (BUN) in both male and female *Scarb2^−/−^* mice was significantly lower than WT (Figure S4C), suggesting that the protein homeostasis may be also disrupted in *Scarb2^−/−^* mice. The decreased phosphorylation level of 4e-bp1, stimulating global protein synthesis, was also in concert with impaired protein catabolism in *Scarb2^−/−^* mice.

The biological function of 4E-BP1 in metabolism has been investigated by disrupting its gene (*Eif4ebp1*) in the mouse. *Eif4ebp1^−/−^* mice manifest markedly smaller white fat pads than wild-type animals, which coincides with our results. Interestingly, knockout males display an increase in metabolic rate and their white adipose tissue contains cells that exhibit the distinctive multilocular appearance of brown adipocytes, expressing higher levels of the uncoupling protein 1 (UCP1) [64]. However, the mRNA expression of several thermogenesis genes, including *Ucp1*, has no differences in *Scarb2^−/−^* mice and control mice (data not shown). On the contrary, another research demonstrated the role of 4E-BPs as a metabolic brake in the development of obesity. They found the combined disruption of *4e-bp1* and *4e-bp2* in mice increased their sensitivity to diet-induced obesity by accelerated adipogenesis driven by increased expression of C/ebpδ, C/ebpα, and Pparγ coupled with reduced energy expenditure, reduced lipolysis and greater fatty acid reesterification in the adipose tissue of *4e-bp1* and *4e-bp2* double KO mice [65]. Thus, the role of 4E-BP1 in adipogenesis or metabolism needs extensive research.

The normal mitochondrial activity is crucial to the function of adipose tissue [66]. BAT contains numerous mitochondria, which exhibit high oxidative capacity and highly express UCP1, which increases thermogenesis by inducing a leak of mitochondrial proton. Although WAT contains fewer mitochondria than BAT, it has been reported that adipocyte differentiation is accompanied by an expansion of mitochondrial mass in WAT [67,68,69]. The major function of mitochondria is energy generation by producing ATP. The whole process depends on the oxidative phosphorylation (OXPHOS) complexes comprised of the electron transport chain (Complexes I–IV) and the ATP synthase (Complex V). The proteins involved need the coordinated interaction between nuclear and mitochondrial genomes. The mitochondrial genome produces only 13 proteins, but they are key components of the respiratory chain. Mitochondrial transcription factor A (TFAM), encoded in the nuclear and present in mitochondria, plays multiple roles in regulating mtDNA replication and transcription to support mitochondrial respiratory function [70,71,72]. Decreased levels of mtDNA and TFAM with impaired mitochondria have been reported in many neurodegenerative diseases, including PD, Alzheimer’s disease as well as in aging [73,74]. Overexpressed TFAM in muscle has been reported to enhance fatty acid (FA) oxidation and reduce the accumulation of fat and incomplete metabolites of FA oxidation, which were beyond the direct effects of TFAM as a transcription factor of mitochondrial biogenesis [75]. Knockdown of TFAM has been shown to significantly decrease mitochondrial respiration and increase glycolysis in colon cancer cells [76]. However, consistent with our observation, interference with Tfam in adipocytes results in mitochondrial respiratory chain dysfunction, reflected by decreased basal oxygen consumption and mitochondrial ATP synthesis [77]. TFAM deletion in the adipose tissue has been reported to increase energy expenditure through decreased complex I activity and increased uncoupling and protect mice against obesity and insulin resistance [78]. Thus, the role of TFAM in regulating mitochondrial respiration and metabolism is tissue specific. In adipocytes, the defect of OXPHOS results in increase energy expenditure and less lipid accumulation.

Adipose tissue is not only an energy storage organ, but also an active and dynamic endocrine organ, which can synthesize and secrete several adipokines [79]. The levels of adipokines are linked to specific metabolic states and have the potential to mediate many signaling cascades, thus they play a critical role in the metabolic homeostasis of the system [80,81]. Lower leptin levels, as well as lower adiponectin levels, have been reported in different LSDs patients with a paucity of adipose storage [82,83]. However, there are also researchers who have reported that the plasma adiponectin, ghrelin and leptin levels in LSD patients are not significantly different from those in the controls [84,85,86,87]. We examined the expression level of adipokines in adipose tissues by real-time quantitative PCR and found that the expression levels of several adipokines (*Leptin, Adiponectin, Chemerin, Rbp4, Fstl1 and Resistin*) are comparable in WT and *Scarb2^Adipoq-cre^* mice (Figure S4E–G). It indicates that lysosomal dysfunction in adipose tissues may not affect adipokines in our study.

In addition to its role as a GCase receptor, SCARB2 belongs to the class B scavenger receptor family that also includes cluster determinant 36 (CD36) and scavenger receptor class B, type 1 (SR-B1). CD36 and SR-B1 are primarily receptors for fatty acids (FAs) and high-density lipoproteins (HDL), respectively, thus they play important roles in lipid metabolism [88,89,90]. Furthermore, SCARB2 was recently reported to be related to lipid metabolism. Conrad et al. defined a dimer structure of SCARB2 with bound phospholipid and cholesterol molecules by biophysical and cellular studies [19]. Another team also reported that the cavity in the luminal domain of SCARB2 can bind and deliver exogenous cholesterol to the lysosomal membrane and later to lipid droplets. Depletion of SCARB2 alters SREBP-2-mediated cholesterol regulation, as well as LDL-receptor levels [20]. Cholesterol does not provide energy directly to the body, but it plays many important functions in our body which are related to metabolism [91]. Although, in this study we did not find any change in cholesterol metabolism in vivo, it does not exclude the role of cholesterol in the regulation of body metabolism in *Scarb2^−/−^* mice. Cholesterol helps the liver create bile acids, which is used by the body to digest consumed food. It has become clear that bile acids are not only digestive detergents but also hormones involved in the regulation of various metabolic processes [92]. Whether the level of bile acids changes in *Scarb2^−/−^* mice will be investigated in the future.

*SCARB2* has also been confirmed as the causative gene of the progressive myoclonic epilepsies (PMEs). As reported before, Scarb2-deficient mice, which lack C-terminal of SCARB2, are characterized by the development of deafness, a unilateral or bilateral hydronephrosis, proteinuria, and a peripheral demyelinating neuropathy [93]. This study mentioned that homozygous mutant and heterozygous mice did not exhibit differences in growth, weight development and fertility, but no data are provided. Our *Scarb2^−/−^* mice showed significantly lower body weight compared to WT mice. This may be due to the different knockout strategies. In our *Scarb2^−/−^* mice, we also found the phenotype of hydronephrosis (Figure S4D). We also monitored the muscular development of *Scarb2^−/−^* mice by the grip strength test, both male and female *Scarb2^−/−^* mice showed skeletal muscle dysfunction compared to WT mice (Figure S4B). Currently, it is believed that the pathogenesis of PME caused by the SCARB2 gene mutation is mainly related to the reduced expression of SCARB2 protein, the abnormal sub-location and the effect of SCARB2 binding to β-GCase. Thus, the deficiency of SCARB2 leads to a pathophysiological process that appears to involve primarily both the brain and the kidneys [94,95]. Whether changes in brain, muscle and kidney may also contribute to abnormal body metabolism need to be further investigated.

Together, the deficiency of SCARB2 is not only associated with diseases such as GD and PD, but also may impair the energy metabolism, which leads to abnormal body weight. It will be worthwhile to investigate the relationship between SCARB2 and metabolic abnormalities because normal physical function is essential for patients to overcome diseases and the treatments targeting SCARB2 could partially rescue associated diseases.

## 4. Materials and Methods

### 4.1. Animals

*Scarb2^−/−^* mice were provided by professor Liguo Zhang from the Institute of Biophysics, Chinese Academy of Sciences. The *Scarb2^−/−^* mice were generated by CRISPR/Cas9 system which deletes 14bp in exon 1 and form a frame shift mutation. The conditional knock out (CKO) *Scarb2^−/−^* mice were provided by the Nanjing Biomedical Research Institute of Nanjing University and generated via CRISPR/Cas9 system. The sg RNA direct Cas9 endonuclease cleavaged in intron 1–2 and intron 2–3 of the *Scarb2* gene and created a DSB (double-strand break). Such break was repaired by donor-mediated homologous recombination and resulted in LoxP sites inserted in intron 1–2 and intron 2–3, respectively, by homologous recombination. Exon 2 was floxed by loxP sites and removed via Adiponectin-cre in *Scarb2^Adipoq-cre^* mice. Mice were maintained in an Association for Assessment and Accreditation of Laboratory Animal Care International-accredited specific pathogen-free (SPF) animal facility. Animal welfare and experimental procedures were approved by the Animal Care and Use Committee of the Model Animal Research Center, Nanjing University.

### 4.2. Cell Culture 

Mouse embryonic fibroblasts (MEFs) were generated from 13.5-day-old embryos obtained from *Scarb2*^+/−^ mice. Briefly, after dissection of head and visceral organs for genotyping, embryos were minced and trypsinized for 20 min at room temperature. Embryonic fibroblasts were then plated and maintained in Dulbecco’s modified Eagle medium (DMEM) with 10% fetal bovine serum (FBS), 100 U/mL penicillin and 100 μg/mL streptomycin at 37 °C in an atmosphere of 5% CO_2_. Genotype analysis was performed to determine cells carrying a homozygous null mutation for *Scarb2*. All experiments were performed with wild-type and *Scarb2^−/−^* MEFs between 0–5 passages. All cell culture reagents are from Thermo (Waltham, MA, USA) 

### 4.3. Isolation of Pre-Adipocytes Cells and Adipocyte Differentiation

Subcutaneous fat tissues were harvested from 10-day-old mice and cut into small pieces with scissors, following incubation in adipose isolation buffer containing 1 mg/mL collagenase (Wako Pure Chemical Industries, Osaka, Japan) for 25 min at 37 °C with gentle shaking. Filter through Cell Strainer (40 µm). Pre-adipocytes were collected as a pellet by centrifugation at 300× *g* for 5 min at 4 °C. Remove supernatant and resuspend pellet in 1 mL basic medium (DMEM, 10% FBS, 1% PS, 50 µg/mL Gentamicin, 25 µg/mL Sodium ascorbate). For adipocyte differentiation, 2-day-postconfluent cells (day 0) were treated with a basic medium supplemented with 850 nanoM Insulin, 0.5 microM Dexamethasone, 250 microM IBMX, 1 microM Rosiglitazone for 2 days (all from Sigma, St. louis, MO, USA). The medium was renewed after 2 days (day 2) with a basic medium supplemented with 160 nm Insulin. The medium was renewed every 2 days with basic medium from day 4. During differentiation, we took pictures of adipocytes and harvested them on day 0, 2, 4, 6 and 8, respectively. After 6 days of differentiation, Oil Red O staining was performed to monitor the intracellular lipid accumulation on day 6 and day 8, respectively.

### 4.4. Histology

WAT, BAT and the liver were isolated and fixed in 4% paraformaldehyde (PFA, Sigma) and processed for HE staining. Sections of 5 μm from the paraffin-embedded tissues were stained with HE for histology analysis. For Oil Red O staining, BAT and liver tissues were fixed in 4% PFA overnight and incubated in 30% sucrose before being cryoembedded and sectioned by Leica. To visualize lipid accumulation, cells were stained with Oil Red O [96]. Briefly, cells were washed with phosphate-buffered saline (PBS), fixed with 4% (w/v) formaldehyde for 30 min and stained with Oil Red O for 15 min using a 60:40 (*v/v*) dilution in water of a 0.5% stock solution (in isopropanol). Cells were then washed once with 60% isopropanol and twice with PBS.

### 4.5. Food Intake

Successive food consumption was calculated using the formula: [(Food mass) on Day (N)—(Food mass) on Day (N+1)], and values were normalized for the number of mice per cage. The same number of mice per cage were set to minimize the impact of housing density on food consumption.

### 4.6. Energy Expenditure and Body Composition Analysis

Body composition (fat and lean mass) analysis was performed on anesthetized mice using dual-energy X-ray absorptiometry (DEXA). For the measurement of energy expenditure, food intake and locomotor activity, the mice were placed in CLAMS (Comprehensive Lab Animal Monitoring System, Columbus Instruments, Columbus, USA) cages that housed in an environmental chamber set at 24 °C. After acclimatization for 1 day, data on oxygen consumption, locomotor activity and food intake were collected every 32 min. Energy expenditure is evaluated through indirect calorimetry by measuring oxygen consumption with an open flow respirometric system. An activity and food and water intake monitoring system can also be integrated into the set up in order to investigate circadian pattern and behaviour. Total activity can be derived from the number of fine movement (e.g., grooming behaviors).

### 4.7. Bomb Calorimetry

Fecal samples were collected and lyophilized to obtain dried mass. Approximately, 200 mg of dried stool was pressed into a pellet using a pellet press. Gross energy content was measured using a semimicro oxygen bomb (IKA 2000, Wilmington, NC, USA) in an isoperibol calorimeter. 

### 4.8. Plasmids

Complementary DNAs (cDNAs) for mouse *Scarb2* cDNA were amplified from reverse-transcribed cDNAs of mouse tissues. The *Scarb2* cDNAs were inserted into the pCDNA5-HA vector linearized with *SmaI* and *NotI* for transient expression in *Scarb2^−/−^* MEFs. All plasmids were verified by DNA sequencing.

### 4.9. Antibodies and Reagents

The antibody against Phospho-p70S6kinase (sc-8416) was purchased from Santa Cruz (Santa Cruz, CA, USA), the antibody against P70S6K (611260) was purchased from BD Biosciences Pharmingen (San Jose, CA, USA), the antibody against phospho-4E BP1 (Ser65) (#9451), 4EBP1 (no. 9452), mTOR (no. 2972), phospho-mTOR (Ser2448) (no. 2971), phospho-mTOR (Ser2481) (no. 2974) and HA-Tag (no. 3724) were purchased from CST (Danvers, USA), the antibody against GAPDH (G8795) was purchased from Sigma. The antibody against Tfam (sc-166965) was purchased from Santa Cruz. Inhibitors of protein trafficking EXO1 (HY-112670) and Brefeldin A /BFA (HY-16592) was purchased from MCE (Shanghai, China). Human LIMPII/SR-B2 Antibody (AF1966) was purchased from R&D (Minneapolis, MN, USA). β Actin (I102) polyclonal antibody (AP0060) was purchased from Bioworld Technology, Inc (Nanjing, China). All the primary antibodies were diluted in a ratio of 1:1000. Secondary antibodies, anti-mouse IgG and anti-rabbit IgG, were purchased from Sigma-Aldrich. All the secondary antibodies were diluted in a ratio of 1:10,000.

### 4.10. Western Blotting

The total cell lysate was collected on ice using lysis buffer (50 mM Tris–HCl, pH 7.4, 150 mM NaCl, 1% Nonidet P-40, 0.1 mM EDTA, 1 mM dithiothreitol and protease inhibitors, including phenylmethylsulfonyl fluoride, Na3VO4 and NaF, plus cocktail protein inhibitor). The concentration of protein samples was determined by the Bradford bioassay—Bradford protein assay kit (Sangon, Shanghai, China). Protein samples were electrophoresed in suitable SDS-PAGE gels and transferred in low temperature to PVDF membranes (Amersham Bioscience, Amersham, UK). Fat-free milk (5%) was used to block blots at room temperature for 1 h, then the blots were incubated with primary antibody overnight at 4 °C. After being washed with Tris-buffered saline and Tween 20 (TBST), the blots were incubated with corresponding secondary antibody for 1 h at room temperature. Proteins were visualized using enhanced chemiluminescence substrate (Tanon, Shanghai, China) and then quantified using a Tanon Chemiluminescent Imaging System.

### 4.11. Real-Time Quantitative PCR Analysis

RNA was extracted from tissues or pre-adipocytes during differentiation by RNAiso Plus (TaKaRa, Shiga, Japan) according to accessary protocol. An HiScript III RT SuperMix + gDNA wiper (Vazyme R323-V10.1, Nanjing, China) was used to synthesize first-strand complementary DNA from equivalent amounts of RNA. Then, quantitative RT-PCR was performed with ChamQTM SYBR^®^ qPCR Master Mix (Vazyme Q311-V9.1). Relative standard real-time PCR was performed on the Roche Light Cycler instrument. Relative gene expression was calculated by the hyperbolic method and was normalized to the expression of the housekeeping gene *Rplp0* (alias *36B4*). The primer sequences are listed in Table 1 and Table S1.

### 4.12. Transmission Electron Microscopy

Fresh WAT tissues from mice were cut into pieces and were immediately fixed in 2.5% glutaraldehyde (SIGMA) for 2 h at room temperature in the dark followed by incubation at 4 °C. The pieces were mailed to the Servicebio Company at 4 °C and follow-up procedures were finished by Servicebio in Wuhan, China.

### 4.13. Seahorse Extracellular Flux Assays 

Measurement of oxygen consumption rate (OCR) and extracellular acidification rate (ECAR) of cells during a mitochondrial stress test was performed using a Seahorse XF-24 Flux Analyzer (Seahorse Biosciences, North Billerica, MA, USA). In brief, cells were seeded in DMEM supplemented with 10% FBS, 100 U/mL penicillin and 100 μg/mL streptomycin 24 h before assay. One hour before assay the media was changed to conditional medium (culture medium without FBS and sodium bicarbonate) and the cells were placed in a 37 °C incubator without CO_2_ buffering. OCR was measured after injection of the following 3 compounds: 1 microM oligomycin, 0.5 microM FCCP, 1 microM antimycin A and rotenone. ECAR was measured after injection of the following 3 compounds: 10 mM glucose, 1 microM oligomycin, 50 mM 2-deoxy-D-glucose (2-DG). Upon completion of the Seahorse XF24 Flux analysis, cells were lysed to calculate the protein concentration using the Bradford method. The results were normalized based on the total amount of proteins in each well.

### 4.14. Lysosomes Detection

LysoTracker™ Green DND-26 (Invitrogen™, L7526, Thermo) was used to mark lysosomes in adipocytes. The adipocytes were incubated with 100 nanoM DND-26 for 30 min at 37 °C. Image- Pro Plus was used to analyze and calculate the diameter of lysosomes. WT and *Scarb2^−/−^* adipocytes each has at least three pictures in each experiments. Results are expressed as mean ± SEM from three independent experiments.

### 4.15. Statistics

The GraphPad Prism software (version 6.01, GraphPad Software, San Diego, CA, USA) was used to analyze and plot all data. Statistical analysis was performed with Student’s t-test for pairwise comparisons and one-way ANOVA for multiple comparisons. *p*-values (<0.05) indicated a significant difference. All values were expressed as the mean ± SEM.

## Figures and Tables

**Table 1:** Summary of qRT-PCR primers.

Primer Name	Forward Sequence	Reverse Sequence
*36B4*	TAAAGACTGGAGACAAGGTG	GTGTACTCAGTCTCCACAGA
*C/EBPα*	CAAGAACAGCAACGAGTACCG	GTCACTGGTCAACTCCAGCAC
*PPARγ*	TCGCTGATGCACTGCCTATG	GAGAGGTCCACAGAGCTGATT
*Ap2*	AGCTGGTGGTGGAATGTGTT	AATTTCCATCCAGGCCTCTT
*Tfam*	GGAATGTGGAGCGTGCTAAAA	ACAAGACTGATAGACGAGGGG
*Scarb2*	AGAAGGCGGTAGACCAGAC	GTAGGGGGATTTCTCCTTGGA

## Data Availability

Data available on request due to restrictions, e.g., privacy or ethical.

## Supplementary Materials

The supporting information can be downloaded at: https://www.mdpi.com/article/10.3390/ijms23158634/s1.
