# Supplementary material for: The Deficiency of SCARB2/LIMP-2 Impairs Metabolism via Disrupted mTORC1-Dependent Mitochondrial OXPHOS
Source: Int J Mol Sci. 2022 Aug 3;23(15):8634. doi: 10.3390/ijms23158634 (PMC9368982; doi:10.3390/ijms23158634)
Supplement: Supplementary file 1 [file ijms-23-08634-s001.zip › ijms-1842210-supplementary.pdf]

International Journal of
Molecular Sciences

Supplemental Materials and Methods

Clinical blood chemistry

The appropriate volume of blood required (160-200 µl of plasma) was collected in gel tube containing lithium Heparin with the relevant blood collection procedure .Time of day for collection is in the morning, starting no earlier than 07:30. Keep whole blood samples in a bag on wet ice until centrifugation. Centrifuge for 10 minutes at 5000 x g in a refrigerated centrifuge set at 8 °C. The plasma samples are analyzed by auto clinic analyzer (HITACHI 7020, Japan Care Co., Ltd. Japan).

Glucose and Insulin Tolerance Tests

For glucose and insulin tolerance tests, mice were intraperitoneally injected with glucose (2 g/kg of body weight) after 16h fast or insulin (0.75 U/kg of body weight) after 6 h fast, and blood glucose was measured at regular intervals using tail blood.

ELISA

The level of corticosterone (CORT) in plasma of mice was evaluated by ELISA according to the manufacturer's protocol. A Mouse CORT ELISA Kit (Cat# SU-B20546, Jining Shiye) was used.

Grip strength test

The grip strength test measured the maximal muscle strength of mouse forelimbs and hind limbs by equipment Bioseb G3 (Chaville, France). Pull the mouse back by its tail while ensuring the torso remains parallel to record the maximal grip strength. Measure forelimb-strength only and the combined forelimb/hindlimb grip strength. Each experiment was repeated three times in succession.

Seahorse extracellular flux assays

Measurement of oxygen consumption rate (OCR) and extracellular acidification rate of cells during a mitochondrial stress test was performed using a Seahorse XF-24 Flux Analyzer (Seahorse Biosciences). In brief, cells were seeded in DMEM supplemented with 10% FBS, 100 U/ml penicillin and 100 µg/ml streptomycin 24 h before assay. One hour before assay the media was changed to conditional medium (culture medium without FBS and sodium bicarbonate) and the cells were placed in a 37 °C incubator without CO2 buffering. Oxygen consumption rate (OCR) was measured after injection of the following 3 compounds: 1 microM oligomycin, 0.5 microM FCCP, 1 microM antimycin A and rotenone. Extracellular acidification rate (ECAR) was measured after injection of the following 3 compounds: 10 milliM glucose, 1 microM oligomycin, 100 milliM 2-deoxy-D-glucose (2-DG). Upon completion of the Seahorse XF24 Flux analysis, cells were lysed to calculate the protein concentration using the Bradford method. The results were normalized based on the total amount of proteins in each well.

Real-time quantitative PCR analysis

RNA was extracted from cells or tissues by RNAiso Plus (TaKaRa) according to accessary protocol. A HiScript III RT SuperMix +gDNA wiper (Vazyme R323-V10.1) was used to synthesize first-strand complementary DNA from equivalent amounts of RNA. Then, quantitative RT-PCR was performed with ChamQTM SYBR® qPCR Master Mix (Vazyme Q311-V9.1). Relative standard real-time PCR was performed on the Roche Light Cycler instrument. Relative gene expression (Table S1) was calculated by the hyperbolic method and was normalized to the expression of the housekeeping gene *Rplp0* (alias *36B4*).

Table S1. Summary of qRT-PCR primers.

Primer Name	Forward Sequence	Reverse Sequence
36B4	TAAAGACTGGAGACAAGGTG	GTGTACTCAGTCTCCACAGA
Crh	CCTCAGCCGGTTCTGATCC	AGCAACACGCGGAAAAAGTTA
Crhr1	GGGCAGCCCGTGTGAATTATT	ATGACGGCAATGTGGTAGTGC
Pomc-a	ATGCCGAGATTCTGCTACAGT	TCCAGCGAGAGGTCGAGTTT
Nr3c1	AGCTCCCCCTGGTAGAGAC	GGTGAAGACGCAGAAACCTTG
Srebf2	GCGTTCTGGAGACCATGGA	ACAAAGTTGCTCTGAAAACAAATCA
Hmgcr	CTTGTGGAATGCCTTGTGATTG	AGCCGAAGCAGCACATGAT
Leptin	GAGACCCCTGTGTCGGTTC	CTGCGTGTGTGAAATGTCATTG
Adiponectin	GTTCCCAATGTACCCATTCGC	TGTTGCAGTAGAACTTGCCAG
Resistin	AAGAACCTTTCATTTCCCCTCCT	GTCCAGCAATTTAAGCCAATGTT
Fstl1	CACGGCGAGGAGGAACCTA	TCTTGCCATTACTGCCACACA
Rbp4	AGTCAAGGAGAACTTCGACAAGG	CAGAAAACTCAGCGATGATGTTG
Chemerin	GCCTGGCCTGCATTAAAATGG	CTTGCTTCAGAATTGGGCAGT

Statistics

GraphPad Prism software (version 6.01) was used to analyze and plot all data. Statistical analysis was performed with Student's t-test for pairwise comparisons and one-way ANOVA for multiple comparisons. P-values (<0.05) indicated a significant difference. All values were expressed as the mean ± SEM.

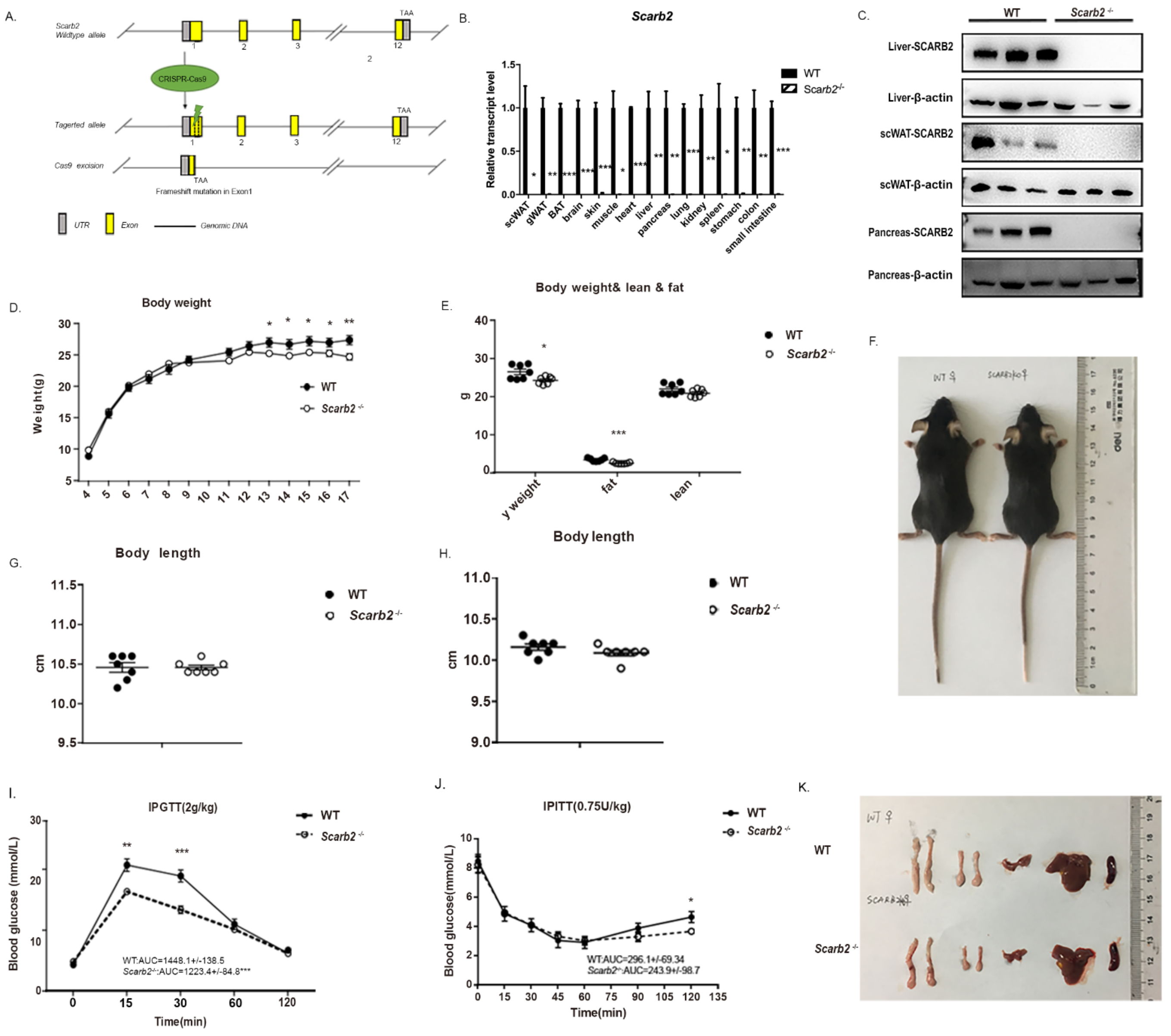

Figure S1. Male *Scarb2*$^{-/-}$ mice show low body weight.

(A) Schematic representation of *Scarb2*$^{-/-}$ mice generated by CRISPR-Cas9.

(B) The expression of *Scarb2* was analyzed by real-time quantitative PCR in different tissues and organs from WT and *Scarb2*$^{-/-}$mouse.

Expression levels of target genes were normalized to *Rplp0* (alias *36B4*) and data presented in (B) were normalized to the mean value of WT group for each tissue or organ. n = 3.

(C) Immunoblot analysis of Scarb2 and β-actin in WT and *Scarb2*$^{-/-}$ mouse tissues (Liver, scWAT, Pancreas) were shown.

(D) Body weights of male WT and *Scarb2*$^{-/-}$mice on a regular chow diet for 4-17 weeks. n = 7.

(E) Body composition of male mice WT and *Scarb2*$^{-/-}$ measured by DEXA after 13 weeks on a regular chow diet. n = 7.

(F) The photo of 6-week-old female WT and *Scarb2*$^{-/-}$ mice.

(G-H) Body length of male **(G)** and female **(H)** WT and *Scarb2*$^{-/-}$ mice after 13 weeks on a regular chow diet. n = 7.
(I) IPGTT of 15-week-old female WT and *Scarb2*$^{-/-}$ mice on a regular chow diet. n = 7.
(J) IPITT of 16-week-old female WT and *Scarb2*$^{-/-}$ mice on a regular chow diet. n = 6.
(K) Representative picture of different organs (scWAT, gWAT, BAT) of 8-week-old female WT and *Scarb2*$^{Adipoq\text{-}cre}$ mice.
DEXA: dual-energy X-ray absorptiometry, IPGTT: Intraperitoneal Glucose tolerance test, IPITT: Intraperitoneal Insulin tolerance test, scWAT: subcutaneous white adipose tissue, gWAT: gonadal white adipose tissue, BAT: brown adipose tissue. Data are represented as mean ± SEM. (*) $P < 0.05$, (**) $P < 0.01$, (***) $P < 0.001$.

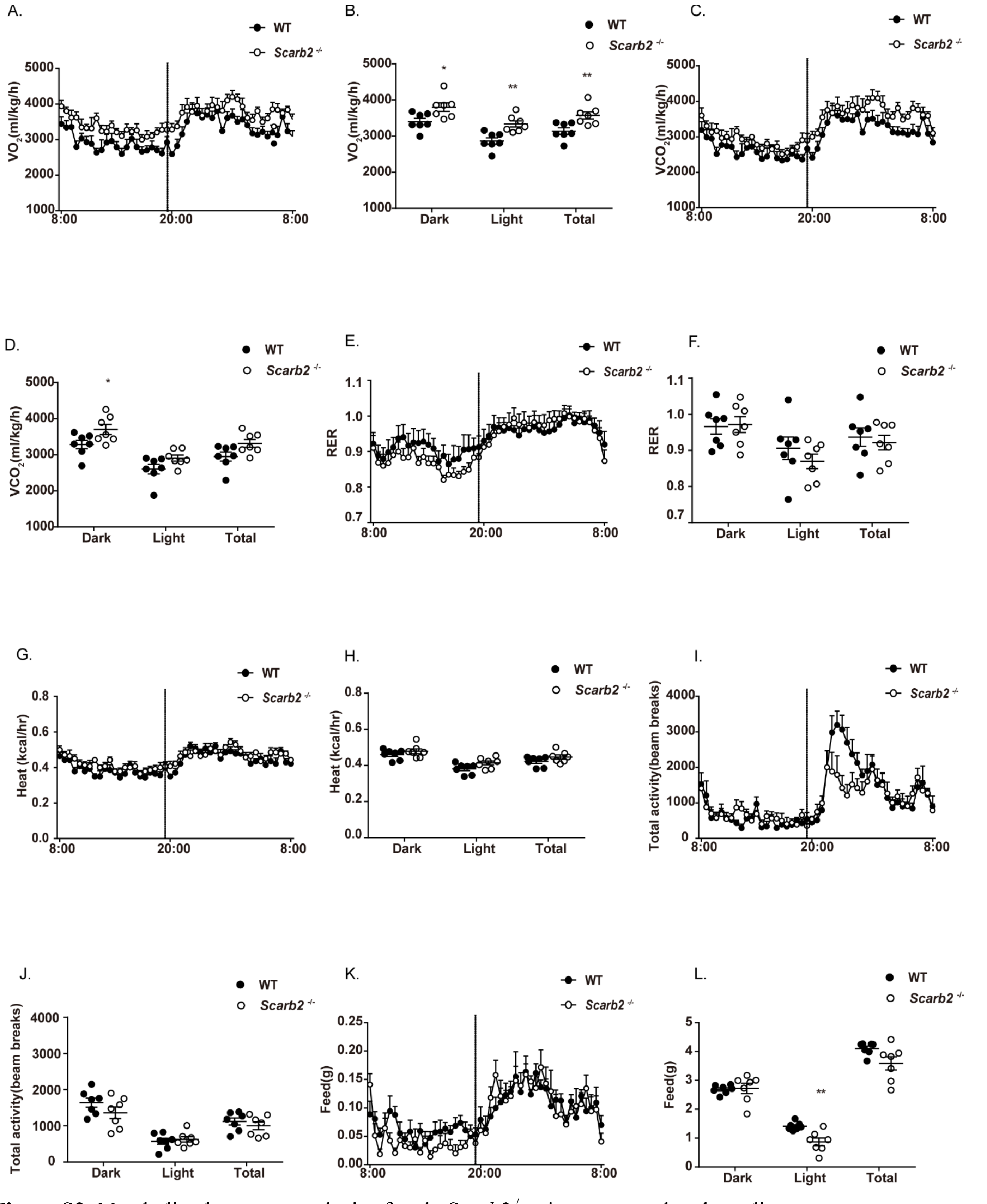

Figure S2. Metabolic phenotype analysis of male *Scarb2*$^{-/-}$ mice on a regular chow diet.
(A-L) Indirect calorimetry of 16-week-old male WT and *Scarb2*$^{-/-}$ mice. **(A, B)** VO_2: Oxygen consumption. **(C, D)** VCO_2: CO_2 generation. **(E, F)** RER: respiration exchange rate, VCO_2/ VO_2.

(G, H) HEAT: heat generation. **(I, J)** Total activity. **(K, L)** FEED: food intake.
The line chart represents real time value of 24 hours for 2 day average.
The column chart represents an average value during the light cycle (8:00 ~20:00) and dark cycle (20:00~8:00).
Data are represented as mean ± SEM. n = 7 for a regular chow diet for all experiments unless otherwise stated. (*) P < 0.05, (**) P < 0.01, (***) P < 0.001.

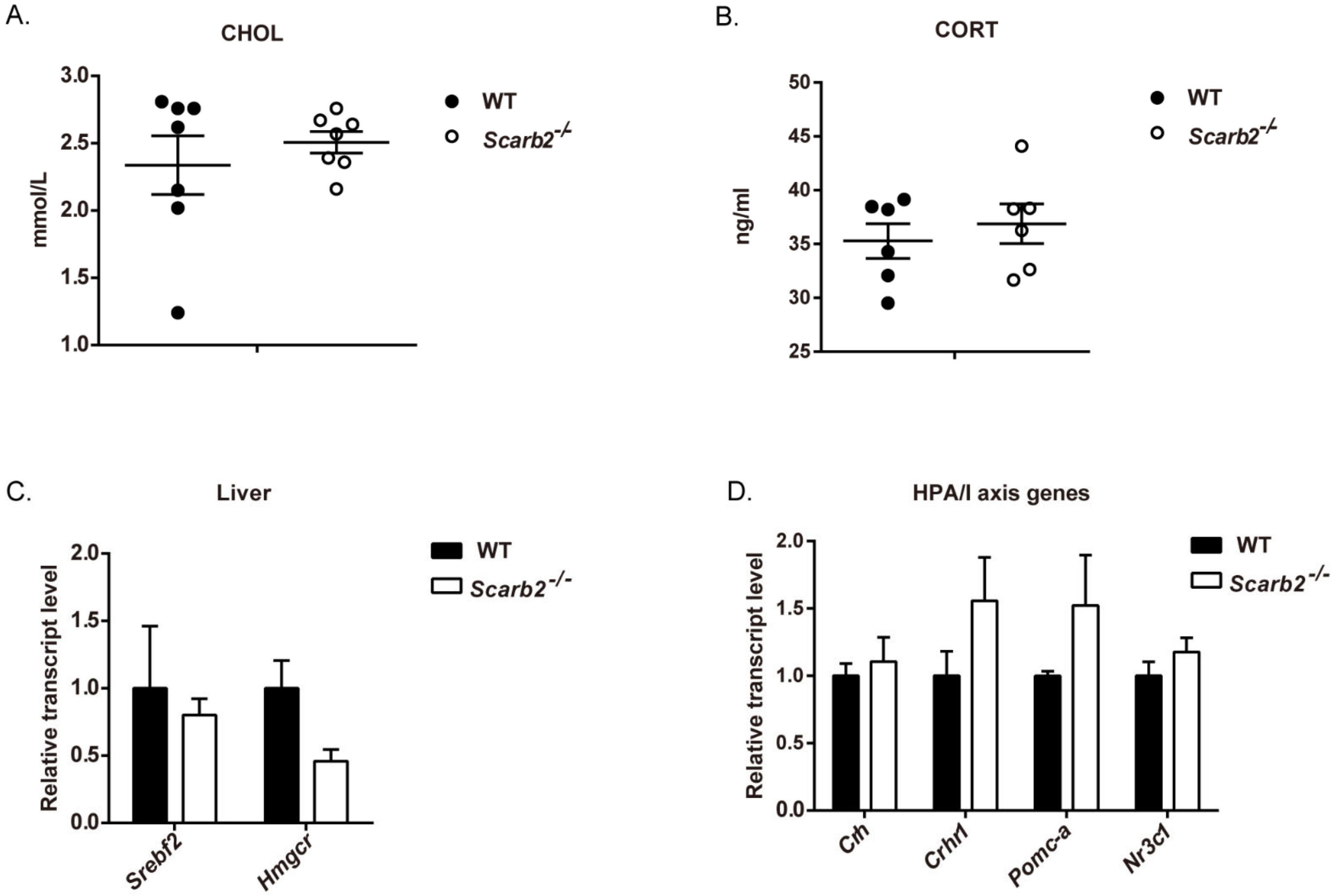

Figure S3. *Scarb2*$^{-/-}$ mice have a normal cholesterol level.
(A) The level of CHOL in plasma of 16-week-old female mice checked by clinical blood chemistry analysis. n = 7.
(B) The level of CORT in plasma of 10-week-old female mice was evaluated by ELISA kit. n = 6.
(C) The expression of *Srebf2* and *Hmgcr* in liver of WT and *Scarb2*$^{-/-}$ mice was evaluated by real-time quantitative PCR. n = 3.
(D) The expression of the HTP/A axis genes: *Crh, Crhr1, Pomc-a, Nr3c1* was evaluated by real-time quantitative PCR. n = 3.
All expression levels of target genes were normalized to *Rplp0* (alias *36B4*). Data presented in (D) were normalized to the mean value of WT group, respectively.
CHOL: total cholesterol, CORT: corticosterone, HTP/A axis: hypothalamic–pituitary–adrenal axis. Data are shown as means ± SEM. (*) P < 0.05, (**) P < 0.01, (***) P < 0.001.

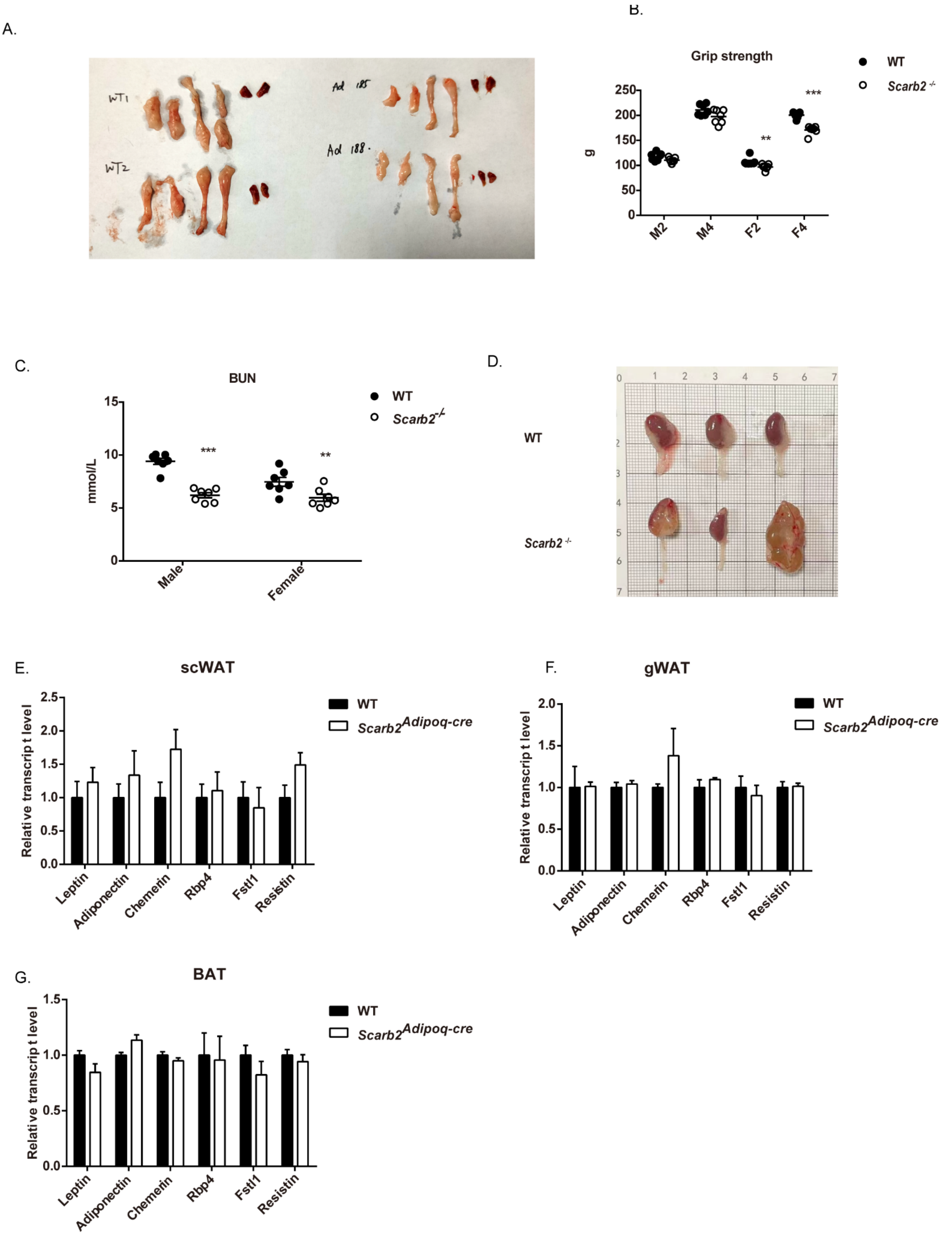
A.
WT1
WT2
Ad 185
Ad 188.
B.
Grip strength
WT
Scarb2 -/-
250
200
150
100
50
0
g
M2
M4
F2
F4
**
***
C.
BUN
WT
Scarb2-/-
15
10
5
0
mmol/L
***
**
Male
Female
D.
WT
Scarb2 -/-
E.
scWAT
WT
Scarb2Adipoq-cre
Relative transcript level
Leptin
Adiponectin
Chemerin
Rbp4
Fstl1
Resistin
F.
gWAT
WT
Scarb2Adipoq-cre
Relative transcript level
Leptin
Adiponectin
Chemerin
Rbp4
Fstl1
Resistin
G.
BAT
WT
Scarb2Adipoq-cre
Relative transcript level
Leptin
Adiponectin
Chemerin
Rbp4
Fstl1
Resistin

Figure S4. The phenotype of *Scarb2*$^{-/-}$ mice and *Scarb2*$^{Adipoq\text{-}cre}$ mice.
(A) Representative picture of different organs from 6-week-old female WT and *Scarb2*$^{-/-}$ mice (scWAT, gWAT, BAT,).
(B) The grip strength test of 9-week-old WT and *Scarb2*$^{-/-}$ mice. M2: male forelimb, M4: male fore and hind limb, F2: female forelimb, F4: female fore and hind limb. n = 7.
(C) The level of BUN in 16-week-old male and female WT and *Scarb2*$^{-/-}$ mice checked by clinical blood chemistry analysis. n = 7.
(D) The photo of kidney of 11-month-old female WT and *Scarb2*$^{-/-}$ mice. n = 3.
(E-G) The expression of several adipokines (*Leptin, Adiponectin, Chemerin, Rbp4, Fstl1, Resistin,*) in scWAT, gWAT and BAT of WT and *Scarb2*$^{Adipoq\text{-}cre}$ mice was evaluated by real-time quantitative PCR. n = 3. Expression levels of target genes were normalized to *Rplp0* (alias *36B4*) and data presented in (E-G) were normalized to the mean value of WT group for each tissue or organ.
Fstl1: follistatin like 1, *Rbp4:* retinol binding protein 4, scWAT: subcutaneous white adipose tissue, gWAT: gonadal white adipose tissue, BAT: brown adipose tissue. BUN: Blood urea nitrogen.Data are shown as means ± SEM. (*) $P < 0.05$, (**) $P < 0.01$, (***) $P < 0.001$.
